# Genome Sequences of Three Strains of *Lactobacillus paracasei* of Different Origins and with Different Cholate Sensitivities

**DOI:** 10.1128/genomeA.00178-15

**Published:** 2015-04-30

**Authors:** Yuh Shiwa, Hotaka Atarashi, Naoto Tanaka, Sanae Okada, Hirofumi Yoshikawa, Akihito Endo, Tatsuro Miyaji, Junichi Nakagawa

**Affiliations:** a Genome Research Center, NODAI Research Institute, Tokyo University of Agriculture, Tokyo, Japan; b Department of Bioindustry, Graduate School, Tokyo University of Agriculture, Hokkaido, Japan; c NODAI Culture Collection Centre, Tokyo University of Agriculture, Tokyo, Japan; d Department of Bioscience, Tokyo University of Agriculture, Setagaya, Tokyo, Japan

## Abstract

We report here the draft genome sequences of three strains of *Lactobacillus paracasei* (NRIC 0644, NRIC 1781, and NRIC 1917) isolated from different sources. The three genomes range from 2.95 to 3.15 Mb with a G+C content of 46% and contain approximately 2,700 protein coding sequences.

## GENOME ANNOUNCEMENT

*Lactobacillus paracasei* is a facultatively heterofermentative lactic acid bacterium which has been found in various sources, including fermented plant materials, fermented milks, and human and animal intestines. Since the species shares similar phenotypic characteristics with its phylogenetic neighbors, i.e., *Lactobacillus casei* and *Lactobacillus rhamnosus*, taxonomy of these three species was thus mixed for some time (1). A conclusion from the Judicial Commission of the International Committee on Systematics of Bacteria led to the solution of this taxonomic problem (2). *L. paracasei* is one of the most characterized lactobacilli, and several beneficial properties related to probiotic application have been reported.

In the present study, three strains of *L. paracasei*, which showed different levels of tolerance to cholic acid, a major component of bile, were genome sequenced to study genetic characteristics related to the bacterial stress response. *L. paracasei* NRIC 1981 and NRIC 1917 were isolated from compost and sugar cane wine, respectively. Strain NRIC 0644 was originally obtained as *L. casei* JCM 1134 (ATCC 393), described as having been isolated from fermented milk. However, our preliminary BLAST analysis indicated that the strain showed low genetic similarities against *L. casei* ATCC 393 (e.g., 81% and 88% sequence similarities with *recA* and *groEL* genes, respectively) but rather high similarities against *L. paracasei* ATCC 334 (99% and 99% similarities with *recA* and *groEL* genes, respectively). NRIC 0644 was therefore identified as *L. paracasei*.

The genomic DNAs of *L. paracasei* strains were extracted with a DNeasy blood and tissue kit (Qiagen). Whole-genome sequencing was carried out by using an Illumina Genome Analyzer II system, with an insert length of about 500 bp. Totals of 6,894,070, 6,499,404, and 20,927,276 reads with averages of 80 to 91 bp were obtained, which yielded 551.5, 591.4, and 1,674.2 Mb sequenced bases (~180-, 200-, and 531-fold coverage). *De novo* assembly using Velvet with parameters optimized by the VelvetOptimizer (3) resulted in 1,436, 141, and 527 contigs with *N*_50_ values of 269,727, 404,054, and 130,768 bp, comprising 3,045,705, 2,950,893, and 3,155,727 bp for NRIC 0644, NRIC 1917, and NRIC 1981, respectively. The mean G+C contents of the strains were 46.4%, 46.3%, and 45.7%, respectively. The assembled sequences were annotated with the Microbial Genome Annotation Pipeline (MiGAP, http://www.migap.org/). tRNAs were detected using the tRNAscan-SE program (4). The draft genomes of NRIC 0644, NRIC 1917, and NRIC 1981 contain 2,712, 2,658, and 2,716 coding sequences (CDSs) and 60, 56, and 61 tRNAs, respectively.

### Nucleotide sequence accession numbers. 

The strains are publicly available in the NODAI Culture Collection Centre, Tokyo University of Agriculture (Tokyo, Japan). The draft of the whole-genome sequencing project has been deposited in DDBJ under the accession numbers BAYM01000001 to BAYM01001436 (NRIC 0644), BAYN01000001 to BAYN01000141 (NRIC 1917), and BAYO01000001 to BAYO01000527 (NRIC 1981).
